# Systematic Analysis of Zinc Finger-Homeodomain Transcription Factors (ZF-HDs) in Barley (*Hordeum vulgare* L.)

**DOI:** 10.3390/genes15050578

**Published:** 2024-05-01

**Authors:** Meng-Di Liu, Hao Liu, Wen-Yan Liu, Shou-Fei Ni, Zi-Yi Wang, Zi-Han Geng, Kong-Yao Zhu, Yan-Fang Wang, Yan-Hong Zhao

**Affiliations:** 1 College of Agriculture, Ludong University, Yantai 264000, Chinaliuhao_1990@126.com (H.L.);; 2 College of Life Science, Ludong University, Yantai 264000, China

**Keywords:** barley (*Hordeum vulgare* L.), genome-wide analysis, *ZF-HD* genes, gene expression

## Abstract

Zinc finger-homeodomain transcription factors (ZF-HDs) are pivotal in regulating plant growth, development, and diverse stress responses. In this study, we found 8 *ZF-HD* genes in barley genome. Theses eight *HvZF-HD* genes were located on five chromosomes, and classified into ZHD and MIF subfamily. The collinearity, gene structure, conserved motif, and *cis*-elements of *HvZF-HD* genes were also analyzed. Real-time PCR results suggested that the expression of *HvZF-HD4*, *HvZF-HD6*, *HvZF-HD7* and *HvZF-HD8* were up-regulated after hormones (ABA, GA3 and MeJA) or PEG treatments, especially *HvZF-HD6* was significantly induced. These results provide useful information of *ZF-HD* genes to future study aimed at barley breeding.

## 1. Introduction

Barley (*Hordeum vulgare* L.) is the fourth largest crop all over the world, which has important applications in food, feed, and brewing [1]. As sessile organism, barley has to suffer various adverse conditions including drought stress, resulting in the loss of yield. Consequently, identification of stress-tolerance genes and breeding stress-tolerance varieties are the key strategies for enhancing the quality and yield of barley.

Zinc-finger homologous domain proteins (ZF-HDs), a kind of plant-specific transcription factors, belong to C2H2 type zinc finger proteins and play vital functions in vegetative growth, flowering, bearing, and stress resistance of plants [2,3,4]. A standard ZF-HD protein comprises a zinc finger structure (ZF) at the N-terminus and a homologous domain (HD) at the C-terminus. The HD domain is a conserved DNA binding domain with about 60 amino acid residues, which participates in plants growth and development by regulating the expression level of downstream target genes [5]. Most HD proteins include other domains that can be involved in interacting with other proteins [6]. The ZF domain comprises two pairs of conserved cysteine and/or histidine residues, which coordinate with a lone zinc ion to configure a finger-like loop structure, thus enhancing the stability of the motif [7]. Zinc finger domain is related to protein nucleic acid recognition and protein-protein interaction [8,9]. Based on conserved motifs and phylogenetic relationships, *ZF-HD* gene family is essentially classified to two subfamilies: ZHD and MIF (mini zinc finger) subfamilies [10]. The ZHD subfamily genes contain both HD and ZF domains [4], and MIF subfamily genes encode ZF-HD proteins with the ZF domains but without the HD domains [11].

ZF-HD protein was initially discovered in *Flaveria trinervia*, that could regulate the expression level of *PEPCase* (phosphoenol pyruvate carboxylase) gene [12]. Subsequently, *ZF-HD* genes was detected in several plant species, such as *Arabidopsis* [4], grapes (*Vitis vinifera*) [13], tomato (*Solanum lycopersicum*) [14], Chinese cabbage [2], Soybean (*Glycine max*) [15], Cucumber (*Cucumis sativus*) [16] and cotton (*Gossypium hirsutum*) [17]. In soybean, *GmZF-HD1* and *GmZF-HD2* respond to pathogen stimulation and bound to the promoter of calmodulin *GmCaM4* gene [15]. In tomato, *ZF-HD* genes were verified to participate in fruit development and stress response [14]. ZHD subfamily gene *AtZHD1* was induced by high salinity, drought and abscisic acid (ABA) in *Arabidopsis* [3]. In addition, *AtZHD1* could interact with NAC transcription factor protein to specifically bind to Early Response to Dehydration (*ERD1*) gene promoter, and improved drought tolerance of *Arabidopsis* [3]. MIF subfamily gene *AtMIF1* participated in multiple hormonal regulation in *Arabidopsis* development process, and overexpressing *AtMIF1* in *Arabidopsis* increased expression level of ABA-responsive genes [10].

*ZF-HD* genes have been investigated in many plant species, but *ZF-HD* family genes in barley have not been identified and analyzed. In this study, we found 8 *ZF-HD* genes in barley genome, and then the physical and chemical properties, chromosomal location, collinearity, exon–intron structure, conserved motif, *cis*-elements and gene expression pattern of *HvZF-HD* genes were analyzed. These results provide useful information of *ZF-HD* genes to future function stud in barley.

## 2. Materials and Methods

### 2.1. Identification of ZF-HD Genes in Barley

The barley protein sequences were downloaded from EnsemblPlant website (https://plants.ensembl.org/index.html) (accessed on 20 January 2024). Hidden Markov Model (HMM) search was used to obtained the proteins with ZF-HD dimerization region (PF04770) in local barley protein sequences [18]. All of the identified HvZF-HD proteins were verified by using SMART (https://smart.embl-heidelberg.de) (accessed on 20 January 2024) and InterPro (http://www.ebi.ac.uk/interpro/) (accessed on 20 January 2024) online services. The ExPASY tools (https://web.expasy.org/protparam/) (accessed on 20 January 2024) was used to predict the physical and chemical parameters of HvZF-HD proteins.

### 2.2. Multiple Sequence Alignment and Phylogenetic Tree Construction

Multiple sequence alignment was performed with ClustalW of MEGA 11 software [19]. The neighbor-joining (NJ) tree was constructed by MEGA 11 with 1000 bootstraps [19]. The ZF-HD protein sequences of *Arabidopsis thaliana*, *Brachypodium distachyon*, *Oryza sativa* and, *Zea mays* were downloaded from the EnsemblPlant website.

### 2.3. Gene Structure and Conserved Motifs Analysis

The exon-intron structure of *HvZF-HD* genes was analyzed by phytozome (https://phytozome.jgi.doe.gov/pz/portal) (accessed on 20 January 2024) and Gene Structure Display Server (GSDS) (https://gsds.cbi.pkuedu.cn/) (accessed on 20 January 2024) website services. The conserved motifs of HvZF-HD proteins were predicted by using Multiple expectation maximization for motif elicitation (MEME) website (https://meme-suite.org/meme/index.html) (accessed on 20 January 2024) [20].

### 2.4. Cis-Elements Analysis

The 2 kb promoter sequences of *HvZF-HD* genes were downloaded from phytozome website (https://phytozome.jgi.doe.gov/pz/portal) (accessed on 20 January 2024), and the *cis*-elements in *HvZF-HD* promoters were predicted with PlantCARE website (http://bioinformatics.psb.ugent.be/webtools/plantcare/html/) (accessed on 20 January 2024) [21].

### 2.5. Chromosome Location and Synteny Analysis

The chromosome location of *HvZF-HD* genes was mapped to chromosome according to barley genome annotation information by using the MapChart [22]. The genome sequences and annotation files of *Arabidopsis*, *G. max*, *H. vulgare*, *O. sativa*, and *Z. mays* were obtained from EnsemblPlant database. The Multiple collinear scanning toolkits (MCScanX) [23,24] and TBtools [25] were used to analyze the collinear relationships between barley and other species.

### 2.6. Transcriptome Analysis

The specific expression of *HvZF-HD* genes in inflorescence (1 cm), inflorescence (5 mm), internode, caryopsis (5 dpa), caryopsis (15 dpa), root (seedling), germinating embryo and shoot (seedling) of barley were obtained from Expression Atlas database (https://www.ebi.ac.uk/gxa/home) (accessed on 20 January 2024).

### 2.7. Plant Materials and Treatments

The seeds of barley cultivar “*MoreX*” were germinated on moist filter paper and grown at 23 °C with a 16 h light/8 h dark photoperiod. For stress and hormone treatments, twenty seedlings grown in hydroponic culture for 5 days were treated with 100 μM GA3 (gibberellin A3), 100 μM ABA (abscisic acid), 100 μM MeJA (methyl jasmonate) and 10% PEG8000 (polyethylene glycol 8000, *w*/*v*) for 36 h, respectively. Then, the shoot tissues were collected, and stored at −80 °C.

### 2.8. RNA Isolation and Real-Time PCR Analysis

SteadyPure Universal RNA Extraction Kit (Accurate Biology) was used to isolate total RNA, and the first-strand cDNA was synthesized by using the Evo M-MLV kit with gDNA clean for qPCR II (Accurate Biology). The specific primers of *HvZF-HD* and *HvActin* genes were showed in Table S1 [26]. Real-time PCR experiments were conducted using TransTaq-T DNA Polymerase (TransGen, Beijing, China) under the following cycling program: initial denaturation at 95 °C for 3 min, followed by 40 cycles of denaturation at 95 °C for 5 s and annealing/extension at 60 °C for 30 s. Real-time PCR experiments were performed in three independent biological replicates and three technical replications to determine the average Ct values. The relative expression levels of *HvZF-HD* genes were calculated by 2^−∆∆CT^ method [27].

## 3. Results

### 3.1. Identification and Characteristic Analysis of ZF-HD Gene Family in Barley

**Figure 1:**
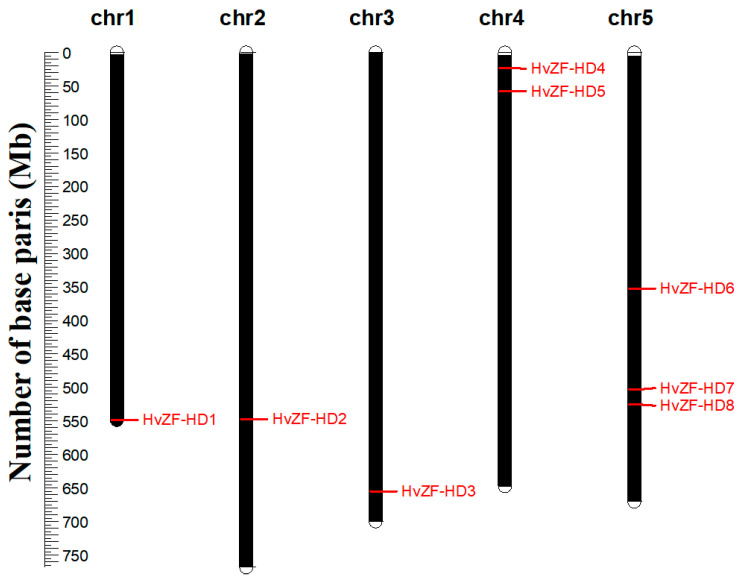
Chromosomal localization of *HvZF-HD* genes.

A total of 8 *ZF-HD* gene were identified by searching for the proteins with ZF-HD dimerization region (PF04770) in local barley protein sequences (Table 1), and SMART and InrerPro online services were used to verified the reliability of these 8 *ZF-HD* genes. Based on chromosome locations of *ZF-HD* genes in barley genome, we named them from *HvZF-HD1* to *HvZF-HD8* (Table 1 and Figure 1). *HvZF-HD1*–*HvZF-HD8* were distributed on 5 chromosomes, i.e., chromosome 1H, 2H, 3H, 4H, 4H, 5H, 5H, and 5H, respectively.

*HvZF-HD* genes encoded polypeptides ranging from 94 to 420 amino acids, with the predicted molecular weights varying from 9.9 to 44.3 kDa (Table 1). The isoelectric point (*p*I) value ranged from 6.9 to 9.5. The calculated grand average of hydrophilic index (GRAVY) ranged from −0.5 to −1.0, indicating that HvZF-HD proteins were hydrophilic proteins (Table 1). Subcellular localization analysis indicated that HvZF-HD proteins were located in the nucleus (Table 1), which confirmed once again that ZF-HD protein was transcription factors.

### 3.2. Evolution and Synteny Analysis of HvZF-HD Genes

**Figure 2:**
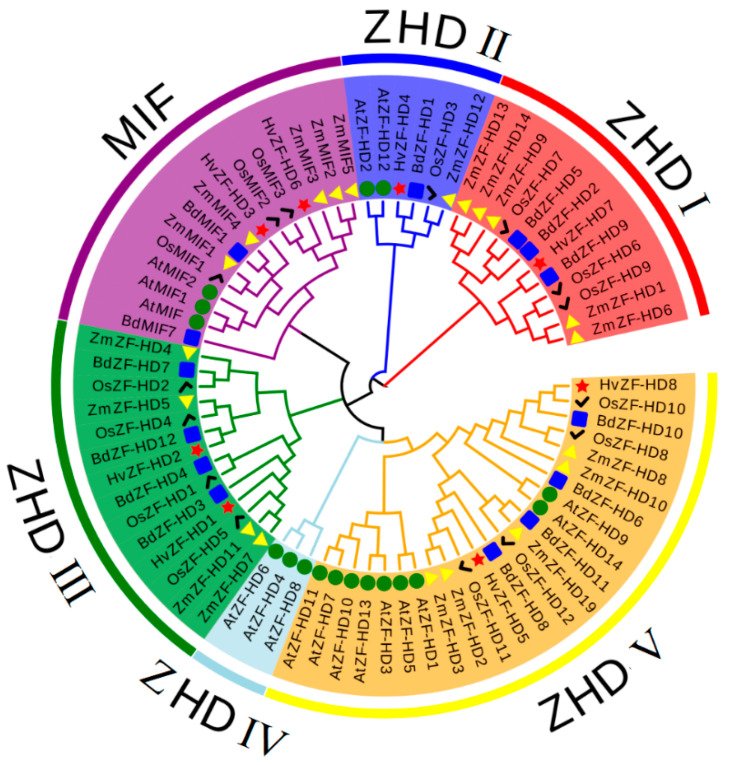
The neighbor-joining (NJ) phylogenetic tree of ZF-HD proteins. The phylogenetic tree was constructed based on ZF-HD protein sequences from *Hordeum vulgare* (Hv), *Brachypodium distachyon* (Bd), *Oryza sativa* (Os) and *Zea mays* (Zm). Different groups of ZF-HD proteins are distinguished by different colors.

The phylogenetic tree was constructed based on ZF-HD protein sequences of *Brachypodium distachyon*, *Hordeum vulgare*, *Oryza sativa*, and *Zea mays* (Figure 2 and Table S2). The results showed that *HvZF-HD* genes were divided into MIF (*HvZF-HD3* and *HvZF-HD6*) and ZHD subfamilies, and the ZHD subfamilies were further classified into five subfamilies, i.e., ZHD I (*HvZF-HD7*), ZHD II (*HvZF-HD4*), ZHD III (*HvZF-HD1* and *HvZF-HD2*), ZHD IV and ZHD V (*HvZF-HD5* and *HvZF-HD8*). Monocotyledons and dicotyledons included both MIF and ZHD subfamily genes, indicating that *ZF-HD* gene family existed before the differentiation of monocotyledons and dicotyledons. However, *ZHD IV* genes were only existed in dicotyledons, suggesting that *ZHD IV* genes appeared after differentiation between dicotyledons and monocotyledons, and evolved separately in dicotyledons.

**Figure 3:**
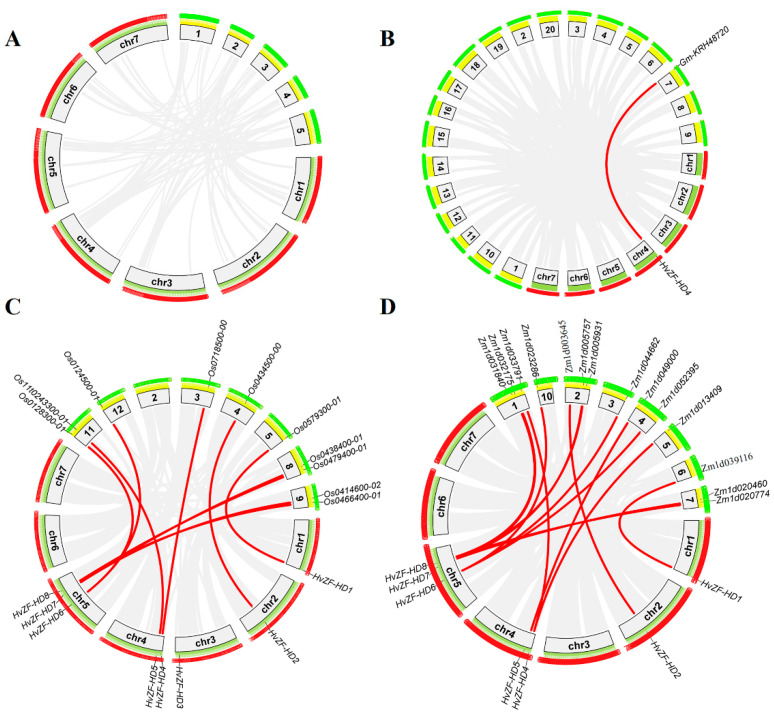
Synteny analysis of *ZF-HD* genes between barley and other plant species. Gray lines in the background indicate the collinear blocks within the barley and other plant genomes, while the red lines highlight the syntenic *ZF-HD* gene pairs. The chromosomes of barley and four other species are painted in different colors, and their names are in the box. (**A**–**D**) Synteny analysis of *ZF-HD* family genes between *Hordeum vulgare* with *Arabidopsis thaliana* (**A**), *Glycine max* (**B**), *Oryza sativa* (**C**) and *Zea mays* (**D**).

We further analyzed synteny between *HvZF-HD* genes in barley with *ZF-HD* genes in other plants, i.e., dicotyledonous plants (*Arabidopsis* and soybean) and monocotyledonous plants (rice and maize) (Figure 3 and Table S3). The results suggested that 0, 1, 10, and 14 orthologous gene pairs were identified between *HvZF-HDs* with other *ZF-HD* genes in *Arabidopsis*, soybean, rice, and maize, respectively (Figure 3). Some *HvZF-HD* genes had at least two orthologous genes with other *ZF-HD* gene in rice and maize, such as *HvZF-HD4*, *HvZF-HD6*, *HvZF-HD7*, and *HvZF-HD8*. These genes might play vital roles in the evolution and expansion of the *ZF-HD* family. The result showed that the genetic relationship of *ZF-HD* gene family was closer with barley in rice and maize than that in *Arabidopsis*, which is consistent with the results of phylogenetic tree (Figure 2). In addition, *HvZF-HD* genes and orthologous genes in other species (soybean, rice and maize) were still highly conserved in a long evolutionary process, suggesting these *ZF-HD* genes might still maintain the similar functions.

### 3.3. Conserved Motifs and Gene Structure Analysis of HvZF-HDs

**Figure 4:**
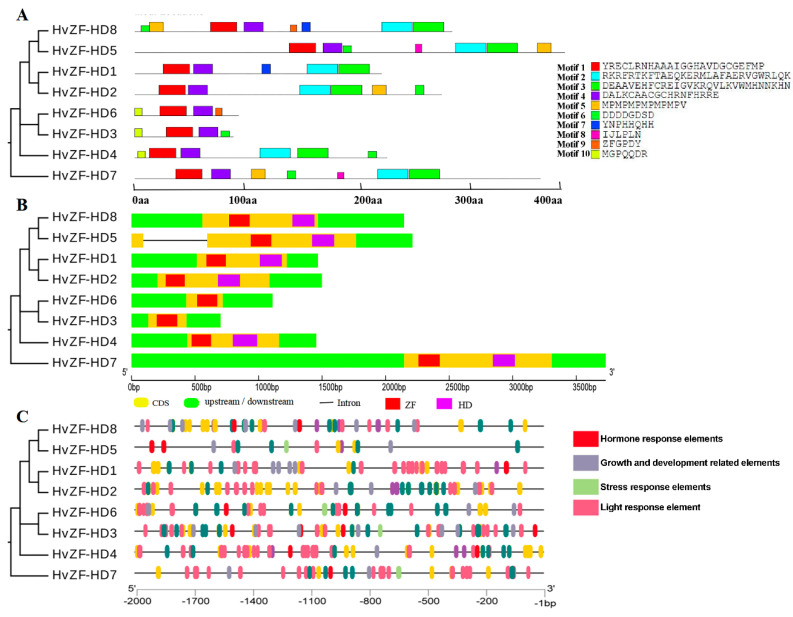
Conserved motifs, gene structures and *cis*-elements analysis of *ZF-HD* genes in barley. The phylogenetic tree was constructed based on HvZF-HD proteins using MEGA 11 software. (**A**) The conserved motifs analysis of the barley HvZF-HD proteins. The motif 1–10 are displayed in different colored boxes. (**B**) Exon-intron structures of the barley *HvZF-HD* genes. Green boxes indicate 5′- and 3′-UTR; yellow boxes indicate exons; and black lines indicate introns. The ZF domain and HD domain are highlighted by red box and purple box, respectively. (**C**) The light response element, hormone response elements, growth and development related elements, and stress response elements are displayed in different colors.

**Figure 5:**
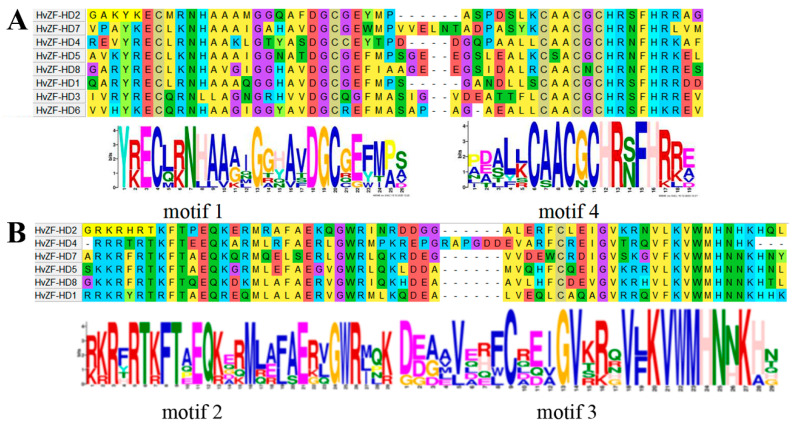
The conserved domain analysis of HvZF-HD protein in barley. (**A**) The ZF domain contain motif 1 and motif 4. (**B**) The HD domain contain motif 2 and motif 3.

To identify the structure characteristic of *HvZF-HD* genes, we analyzed the conserved motifs and exon-intron structures, respectively (Figure 4). Conserved motif analysis showed that 10 motifs were identified in HvZF-HD protein, and all HvZF-HD proteins contained motif 1 and motif 4, which consisted of the core sequence of ZF domain (Figure 4A and Figure 5A). In addition, Motif 2 and motif 3 comprised the typical HD domain (Figure 4A and Figure 5B). The results of conserved domain analysis suggested that the HvZF-HD proteins contained HD domain (HvZF-HD1, HvZF-HD2, HvZF-HD4, HvZF-HD5, HvZF-HD7 and HvZF-HD8) belonged to ZHD subfamily. Meanwhile, HvZF-HD3 and HvZF-HD6, which had no HD domain, belonged MIF subfamily (Figure 4A and Figure 5B).

The exon-intron structure of *HvZF-HD* genes was also analyzed, most *HvZF-HD* genes had no introns except *HvZF-HD5* included one intron (Figure 4B). The difference of intron number indicates that the *HvZF-HD* gene family may have acquired or lost introns in the evolutionary process, and intron number also implies the potential ability of genes to form multiple splices. In conclusion, *HvZF-HD* genes with closer evolutionary relationships had similar gene structures (Figure 4B).

### 3.4. The Cis-Elements Analysis of HvZF-HD Promoters

The *cis*-elements in the gene promoter regions were related to gene expression, thus gene could perform different function in various environmental conditions [28]. The *cis*-elements in the promoter regions of the *HvZF-HD* genes were analyzed by PlantCARE [21], and the *cis*-elements identified were divided into four categories: light response element, hormone response elements, growth and development related elements, and stress response elements (Figure 4C and Table S4). The types and distribution of *cis*-element in the upstream of *HvZF-HDs* were diverse, suggesting that *HvZF-HD* gene family participated in the regulation of multiple signal pathways to respond to environmental stress. Each *HvZF-HD* promoter region contained at least one light response element, indicating that the expression of *HvZF-HDs* might be regulated by light. The diversity of *cis*-element type and number in the promoter of *HvZF-HD* genes showed that *HvZF-HD* genes participated in various hormones and stress response.

### 3.5. Expression Patterns of HvZF-HD Genes in Different Tissues

**Figure 6:**
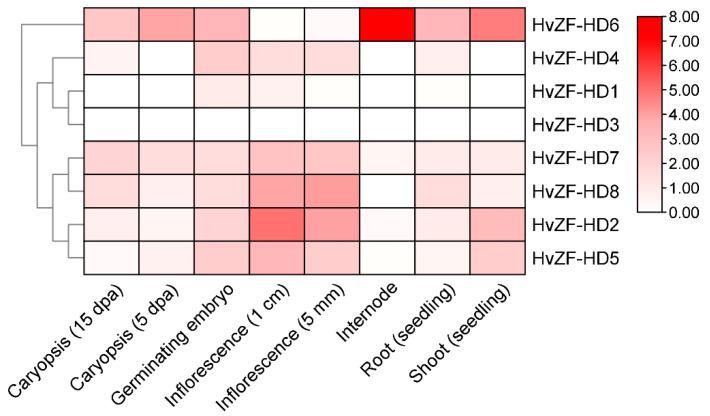
Tissue-specific expression patterns of the *HvZF-HD* genes in *Hordeum vulgare*. The transcriptome data of various tissues in barley were downloaded from Experiments database. The log_2_ of FPKM (fragments per kilobase of exon model per million mapped) values were calculated by RNA-seq data to show the expression levels of the *HvZF-HD* genes in barley.

To identify tissue-specific expression profiles of *HvZF-HD* genes in barley, transcriptome data of inflorescence (1 cm), inflorescence (5 mm), internode, caryopsis (5 dpa), caryopsis (15 dpa), root (seedling), germinating embryo and shoot (seedling) were analyzed. The results showed that the transcript abundance of *HvZF-HD* genes was different in various tissues (Figure 6 and Table S5), suggesting that *HvZF-HD* family genes performed multiple functions in the growth and development of barley. For example, *HvZF-HD6* was highly expressed in internode and shoot tissues, suggesting *HvZF-HD6* might play important roles in vegetative tissues. *HvZF-HD2* and *HvZF-HD8* were highly expressed in both inflorescence, suggesting that these two genes play vital roles in the flowering process of barley (Figure 6 and Table S5). In addition, *ZHD* subfamily genes (*HvZF-HD1*, *2*, *4*, *5*, *7*, and *8*) were higher expressed in inflorescence compared with MIF subfamily genes (*HvZF-HD3* and *HvZF-HD6*) (Figure 6 and Table S5), indicating that the *ZHD* subfamily genes might be involved in regulating flower growth.

### 3.6. Expression Patterns of HvZF-HD Genes after PEG and Hormone Treatments

**Figure 7:**
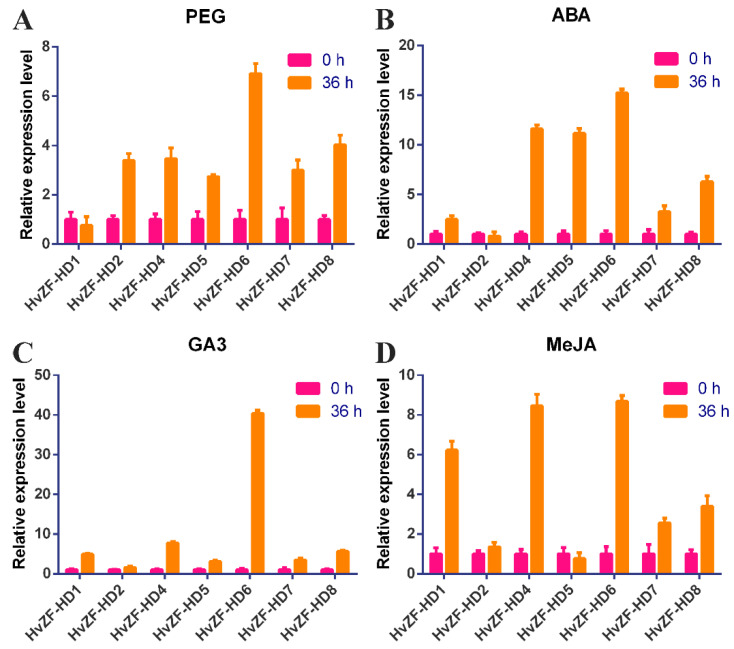
Expression levels of *HvZF-HD* genes after PEG (**A**) and various hormone (ABA, GA3, MeJA) treatments (**B**–**D**) in barley. The expression level of the barley *HvActin* gene was used as the internal control to standardize the RNA samples for each reaction. The values are the mean ± SD from three samples.

To explore the role of *HvZF-HD* genes in barley, the expression levels of seven *HvZF-HD* genes (*HvZF-HD1*–*2*, and *HvZF-HD4–HvZF-HD8*) after PEG and hormone (ABA, GA3 and MeJA) treatments in shoot tissues of barley at the seedling stage were detected by real-time PCR (Figure 7). All genes except *HvZF-HD1* were up-regulated after PEG stress for 36 h (Figure 7A). After ABA treatment, only *HvZF-HD2* gene was down-regulated, and other six genes were highly induced by ABA treatment (Figure 7B). All *HvZF-HD* genes were up-regulated after GA3 treatment (Figure 7C). It is worth noting that the expression level of *HvZF-HD6* was up-regulated (40-fold) at 36 h after GA3 treatment compared to control (Figure 7C). After MeJA treatment, the expression level of *HvZF-HD5* was down-regulated, and other *HvZF-HD* genes were up-regulated Figure 7C,D). In conclusion, most *HvZF-HD* genes were significantly induced after PEG, ABA, GA3 and MeJA treatments, therefore, we speculated that the *HvZF-HD* family might play important roles in stress defense response.

## 4. Discussion

The members of *ZF-HD* gene family have been detected in many plants, but a genome-wide analysis was not performed in barley. Previous studies have reported that ZF-HD family were classified into MIF and ZHD subfamilies [17], our results also confirm these result (Figure 2). Interestingly, the evolutionary relationship of MIF proteins was close with ZHD III proteins (Figure 2), suggesting the MIF proteins might originate from the deletion of the HD domain of ZHD proteins, or ZHD proteins might originate from the HD domain obtained by MIF proteins.

Collinear analysis showed ten orthologous gene pairs were found between barley and rice, fourteen orthologous gene pairs were found between barley and maize (Figure 3 and Table S3). Some *HvZF-HD* genes had two or more orthologous genes, such as *HvZF-HD6* were collinear with three *ZF-HD* genes in maize, which might play crucial roles in the evolution of *ZF-HD* genes. (Figure 3 and Table S3). Moreover, only one orthologous gene pair of *ZF-HD* genes was found between barley and *Glycine max* (Figure 3B). This phenomenon might be caused by separate evolution of *ZF-HD* genes in monocotyledons and dicotyledonous plants.

*ZF-HD* genes regulate many biological processes and play an important role in plant growth, development and stress response [3,29]. The result of tissue-specific expression profiles showed that *HvZF-HD* genes had different expression levels in different barley tissues (Figure 6), thus *HvZF-HD* genes might play various roles in plant growth and development. The *HvZF-HD3* was not detected in barley tissues, suggesting that *HvZF-HD3* might be expressed in specific tissues and environment conditions, or it was a pseudogene. *HvZF-HD* genes were mainly expressed in inflorescence, while most *ZF-HD* genes in *Arabidopsis thaliana* were universally expressed in flower tissue [4], indicating that *ZF-HD* genes might play a role in regulating flower development. Interestingly, the two MIF subfamily genes (*HvZF-HD3* and *HvZF-HD6*) were not expressed in the inflorescence. Although *HvZF-HD6* was not expressed in the inflorescence, it is highly expressed in other tissues, especially in the internodes, indicating that *HvZF-HD6* may play an important role in the vegetative growth of barley. In addition, most *HvZF-HD* genes were significantly induced after PEG, ABA, GA3 and MeJA treatments, suggesting that the *HvZF-HD* genes played vital roles in plant growth, plant growth, development, and stress defense response (Figure 7). These results laid a foundation for future functional studies of *HvZF-HD*s.

## Figures and Tables

**Table 1:** The *HvZF-HD* family genes in barley.

Gene Name	Gene ID	Subfamily	Genomic Position	Gene Length (bp)	CDS Length (bp)	Protein Length (aa)	Molecular Weight (kDa)	*p*I	GRAVY	Subcellular Localization
*HvZF-HD1*	HORVU1Hr1G091470	ZHD II	chr1H:548516561:548517303:+	1467	708	235	25.9	9.5	−1.0	Nucleus
*HvZF-HD2*	HORVU2Hr1G075950	ZHD III	chr2H:547421324:547421478:+	1499	879	292	30.1	7.7	−0.5	Nucleus
*HvZF-HD3*	HORVU3Hr1G096990	MIF	chr3H:654844432:654845132:+	701	300	99	10.8	9.0	−0.6	Nucleus
*HvZF-HD4*	HORVU4Hr1G008360	ZHD IV	chr4H:22781863:22783316:-	1454	723	240	25	7.7	−0.8	Nucleus
*HvZF-HD5*	HORVU4Hr1G015250	ZHD VI	chr4H:58179068: 58180126:-	2211	1263	420	44.3	8.2	−0.6	Nucleus
*HvZF-HD6*	HORVU5Hr1G045580	MIF	chr5H:352394656:352394965:+	1110	285	94	9.9	6.9	−0.5	Nucleus
*HvZF-HD7*	HORVU5Hr1G065740	ZHD I	chr5H:502714705:502714876:+	3732	1161	386	40.5	8.5	−0.5	Nucleus
*HvZF-HD8*	HORVU5Hr1G069730	ZHD V	chr5H:525363232:525364129:-	2145	909	302	31.8	7.1	−0.5	Nucleus

## Data Availability

The public RNA-seq data were obtained from Expression Atlas database (https://www.ebi.ac.uk/gxa/experiments) (accessed on 20 January 2024).

## Supplementary Materials

The following supporting information can be downloaded at: https://www.mdpi.com/article/10.3390/genes15050578/s1, Table S1. Real-time PCR primers of *HvZF-HD*s and the *HvActin* genes; Table S2. ZF-HD proteins used in the phylogenetic tree construction; Table S3. Synteny analysis of *ZF-HD* genes between barley and four species (Arabidopsis, rice, maize and soybean); Table S4. *Cis*-elements analysis of the promoters of *HvZF-HD* genes; Table S5. The expression levels of *HvZF-HD* genes in different tissues of barley.
